# Oxytocin Receptors Regulate Social Preference in Zebrafish

**DOI:** 10.1038/s41598-020-61073-4

**Published:** 2020-03-25

**Authors:** Jenny Landin, Daniel Hovey, Bo Xu, David Lagman, Anna Zettergren, Dan Larhammar, Petronella Kettunen, Lars Westberg

**Affiliations:** 1 Department of Pharmacology, Institute of Neuroscience and Physiology, University of Gothenburg, Gothenburg, Sweden; 2 Department of Psychiatry and Neurochemistry, Institute of Neuroscience and Physiology, University of Gothenburg, Gothenburg, Sweden; 3 Department of Neuroscience, Unit of Pharmacology, Science for Life Laboratory, Uppsala University, Uppsala, Sweden

**Keywords:** Social behaviour, Autism spectrum disorders

## Abstract

With a strong tendency to socialise, the zebrafish is a useful model to study social behaviour, with implications for better treatments of social impairments, for instance in autism spectrum disorders. Although oxytocin is crucial for social behaviour in mammals, the importance of the fish orthologue – isotocin or zebrafish oxytocin (zOT) – for social behaviour in zebrafish is unclear. The aims of this study were firstly, to elucidate the receptor specificity of zOT and the related vasotocin or zebrafish vasopressin (zVP; the orthologue of mammalian vasopressin) and the nonpeptidergic oxytocin receptor antagonist L-368,899, and secondly to investigate if L-368,899 inhibits social preference in zebrafish. The potencies of ligands were evaluated for zOT/zVP family receptors in HEK293 cells. Adult and larval zebrafish were treated with L-368,899 or vehicle and subsequently assessed for social behaviour and anxiety (adults only). The antagonist L-368,899 specifically inhibited the two zOT receptors, but not the two zVP-1 receptors. The antagonist decreased social preference in adult and larval zebrafish. It did not affect anxiety in adults. These results indicate that endogenous zOT, and possibly zVP, is involved in social behaviour in zebrafish via either or both of the two zOT receptors, and show promise for future explorations of the anatomy and evolution of networks underlying social behaviour.

## Introduction

Oxytocin (OT) is a highly conserved neuropeptide, which in slightly different variants, has been found in all investigated vertebrates. The orthologous peptide of some animal groups has been given different names based on their amino acid sequences, for example in birds (mesotocin), and in teleost fishes (isotocin)^1–4^. In mammals, OT has been implicated in the regulation of social behaviour and social cognition as well as in regulation of anxiety^5^. Receptors for OT and its paralogue vasopressin (VP, often called vasotocin in fishes), are distributed in various brain regions associated with stress, anxiety, and social behaviour^6^. Impairments in social behaviour are intrinsic to several neurodevelopmental and neuropsychiatric disorders, including autism spectrum disorder, where these impairments constitute a core feature of the disorder^7^. Intranasally administered OT has been shown to exert effects on various social behaviours in healthy human subjects^8^, and initial clinical studies indicate that OT is a promising treatment candidate for autism spectrum disorders (ASD)^9^. Several studies also indicate that variants in OT-related genes associate with the risk for ASD^10^ and with endophenotypes for ASD^8,11,12^. Hence, it is imperative to closely deduce the mechanisms modulating social behaviour, including that of OT, in order to facilitate the development of effective treatments.

The zebrafish (*Danio rerio*) has emerged as an animal model for biomedical research, especially in developmental studies and drug discovery^13,14^, and has been successfully used to investigate the neural circuitry of several behaviours^15^, such as prey capture^16^ and locomotor control^17^. Furthermore, the zebrafish is also rapidly proving to be a useful model for social neurobiology^18^. Social behaviour is well developed in zebrafish^19^, as apparent in the expression of preference towards conspecifics^20^ as well as the ability to discriminate kin and familiar individuals^21–23^. Adults and larvae (at an age of about 3 weeks) exhibit clear social preference^24–28^. Similar to humans, zebrafish social interaction relies upon vision and, zebrafish display clear responses to visual images of conspecifics^20,29^.

While the physiological and behavioural roles of OT and VP orthologues are established in many other vertebrates, they remain unclear in zebrafish. Similar to mammals, the zebrafish OT orthologue isotocin [herein referred to as zebrafish OT (zOT)] and the VP homologue vasotocin [herein referred to as zebrafish VP (zVP)] are expressed in the preoptic area of the zebrafish hypothalamus^30–32^, from which the nonapeptidergic neurons project to diverse brain areas^31–33^. Whereas mammals have only one OT receptor gene, *OXTR*, the zebrafish has two orthologous receptor genes, OT receptor (*oxtr*) and OT receptor like (*oxtrl*). This is the result of a local gene duplication in the teleost lineage^34^. Gene duplication has been proposed to serve as an important evolutionary mechanism because it generates copies that may evolve new or more specialised functions^35,36^. Moreover, OT and VP are related nonapeptides differing by only two amino acids, and at least in mammals some of the actions of OT are mediated through VP receptors and vice versa^37,38^. In zebrafish (and other ray-finned fishes), evidence indicate that the two nonapeptides play important roles in regulation of social behaviour^39^, but the knowledge regarding the cross-reactivity of the two peptides to their receptors in zebrafish is sparse. This is important to sort out since the zebrafish have the two previously mentioned zOT receptors, as well as two zVP-1 receptors as a result of the teleost fish tetraploidisation^34,40^.

The levels of zOT (as well as zVP) are associated with the outcome of agonistic social interactions^41^. Moreover, zOT has been demonstrated to increase social preference and decrease fear response to a predator in a dose-dependent manner in zebrafish^42^. Furthermore, specific ablation of zOT neurons in the posterior tuberculum at the embryonic stage resulted in reduced shoaling behaviour and reduced social preference in adult zebrafish^43^. Treatment with zOT or the OT receptor agonist carbetocin rescued social deficits in zebrafish following exposure to the glutamate antagonist MK-801, but the OT receptor antagonist L-368,899 did not reverse those deficits^44^.

### Aims

L-368,899 is a non-peptidergic antagonist, previously shown to specifically inhibit the OT receptor in mammals^45,46^. As the specificity, potency, and selectivity were unknown for zOT and zVP, and the antagonist L-368,899, on the two zebrafish receptors for zOT (Oxtr and Oxtrl) and the two receptors for zVP (Avpr1aa and Avpr1ab), the initial aim was to investigate this after expression of the cloned receptors *in vitro*. Subsequently, to increase our understanding of the relation between social behaviour and the zOT receptors in adult and larval zebrafish, we used L-368,899 to block the zOT receptors, and measure the effect on social preference, shoaling behaviour, and anxiety-like behaviour.

## Results

### Ligand potencies at zOT and zVP receptors

**Figure 1:**
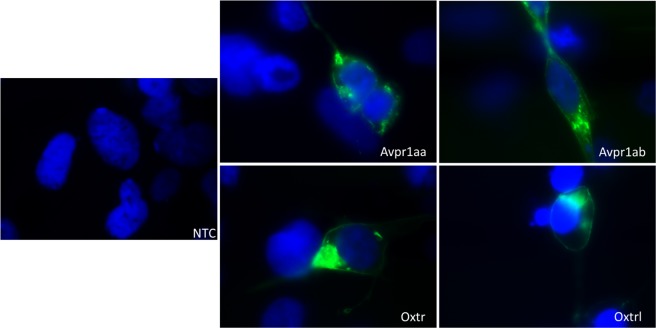
Expression of zebrafish oxytocin and vasopressin receptors in HEK293 cells. Pictures were taken using a 100x objective lens. Blue: DAPI; Green: GFP; NTC: Non-Template Control.

In our *in vitro* studies, the two zOT receptor subtypes and two zVP receptor-1 subtypes were individually expressed in HEK293 cells (Fig. 1). As shown in Table 1, zOT and zVP had much higher potency for Oxtr, Oxtrl, and Avpr1ab receptors compared to Avpr1aa.

**Table 1:** Potencies (EC50) of agonists and the oxytocin receptor antagonist L-368,899 (IC50) in competition with 1 μM zebrafish vasopressin.

Receptors	Oxtr (nM)		Oxtrl (nM)		Avpr1aa (nM)		Avpr1ab (nM)	
Ligands	Mean ± SEM	n	Mean ± SEM	n	Mean ± SEM	n	Mean ± SEM	n
zOT	2.99 ± 0.93	3	3.14 ± 1.10	3	317 ± 79	4	3.52 ± 0.94	3
zVP	11.0 ± 3.0	3	27.0 ± 9.5	4	727 ± 338	3	2.79 ± 1.4	3
OT	47.9 ± 7.9	3	34.8 ± 7.2	3	21.5 ± 5.5	3	42.1 ± 9.7	3
L-368,899	282 ± 74	4	225 ± 31	4	—		—	

The symbol “—” indicates that the potency could not be determined accurately due to binding affinity being too low to compete away the zebrafish vasopressin (zVP). OT = mammalian oxytocin, zOT = zebrafish oxytocin.

SEM = standard error of the mean.

**Figure 2:**
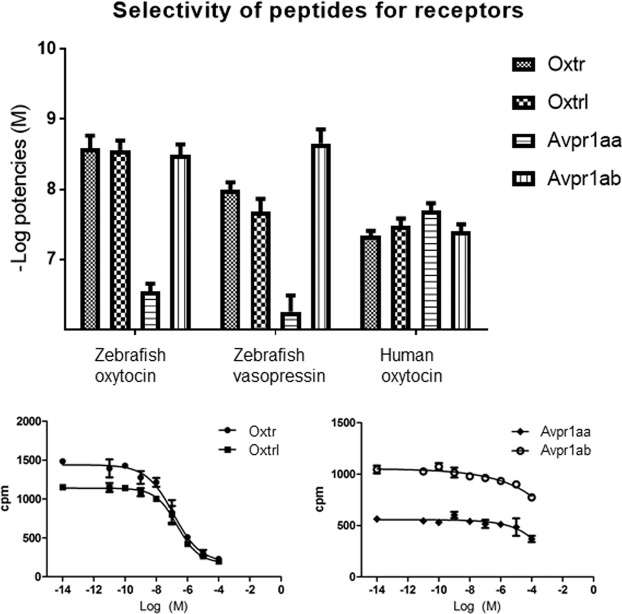
(**a**) Potencies (pEC50 values) of zebrafish oxytocin (zOT), zebrafish vasopressin (zVP) and human oxytocin at zebrafish OT (Oxtr and Oxtrl) and VP receptors (Avpr1aa and Avpr1ab). For p-values, see Results section. (**b**) L-368,899 dose-response curves for Oxtr, Oxtrl, Avpr1aa, Avpr1ab stimulated by zVP. cpm = counts per minute.

To evaluate whether the differences in ligand potencies for the receptors were statistically significant, two-way ANOVA tests (Bonferroni post-tests) were performed for the pEC50 values. zVP’s pEC50 value for Avpr1ab was significantly higher than for Oxtr (p < 0.05) and Oxtrl (p < 0.001). Also, zVP had significantly higher pEC50 values (lower potency) for Oxtr (p < 0.05) and Oxtrl (p < 0.01) compared with zOT. Both zOT and zVP had low potency for Avpr1aa (>317 nM) (Table 1; Fig. 2). On the other hand, mammalian OT had moderate potency at all four receptors (in the interval of 20–50 nM) (Table 1; Fig. 2). The antagonist L-368,899 was able to block zVP activation of both of the Oxtrs (Table 1). In contrast, the antagonist did not block the response of the two zVP receptors, neither Avpr1ab nor Avpr1aa, when stimulated with agonist, not even when the antagonist was present at 1 μM. This suggests that L-368,899 cannot bind and block peptide activation of the two zVP receptors.

### Locomotion in adult zebrafish

There were no significant differences in activity parameters (average distance swum and average velocity) between fish treated with vehicle and L-368,899 during the social preference test (average distance, p = 0.25); average velocity, p = 0.32), the shoaling test (average distance, p = 0.37; average velocity, p = 0.30), or in the novel tank diving test (average distance, p = 0.49; average velocity, p = 0.47) (Table 2).

**Table 2:** Means of outcome variables for all tests.

Test	Behaviour	Measure	Vehicle	L-368,899
Adult social preference	Locomotion	Distance (cm/min)	528 ± 19	496 ± 20
		Velocity (cm/s)	9.1 ± 0.3	5.6 ± 0.3
	Social behaviour	Social zone duration (s/min)	45.9 ± 2.3	34.5 ± 2.4
		Distance to shoal (cm)	11.0 ± 1.8	19.7 ± 1.9
Adult shoaling	Locomotion	Distance (cm/min)	207 ± 15	186 ± 17
		Velocity (cm/s)	3.6 ± 0.2	3.2 ± 0.3
	Social behaviour	Interfish distance (cm)	5.9 ± 0.3	6.7 ± 0.2
		Nearest neighbour distance (cm)	3.3 ± 0.2	3.7 ± 0.1
		Furthest neighbour distance (cm)	8.7 ± 0.5	9.8 ± 0.2
Adult novel tank diving	Locomotion	Distance (cm/min)	186 ± 11	174 ± 14
		Velocity (cm/s)	3.1 ± 0.2	2.9 ± 0.2
	Anxiety behaviour	Distance to bottom (cm)	2.7 ± 0.4	3.1 ± 0.4
		Latency to top zone (s)	327 ± 58	387 ± 81
		Duration in top zone (s/min)	7.0 ± 2.4	8.8 ± 2.4
Larvae social preference	Locomotion (AC)	Distance (cm/min)	249 ± 12	265 ± 14
		Velocity (cm/s)	0.28 ± 0.01	0.30 ± 0.02
	Social behaviour	Social Preference Index (AC)	0.06 ± 0.04	0.00 ± 0.05
		Social Preference Index (SC)	0.18 ± 0.05	0.01 ± 0.06

All values are means ± standard error of the mean. AC: acclimation phase. SC: social cue phase.

### Social preference in adult zebrafish

**Figure 3:**
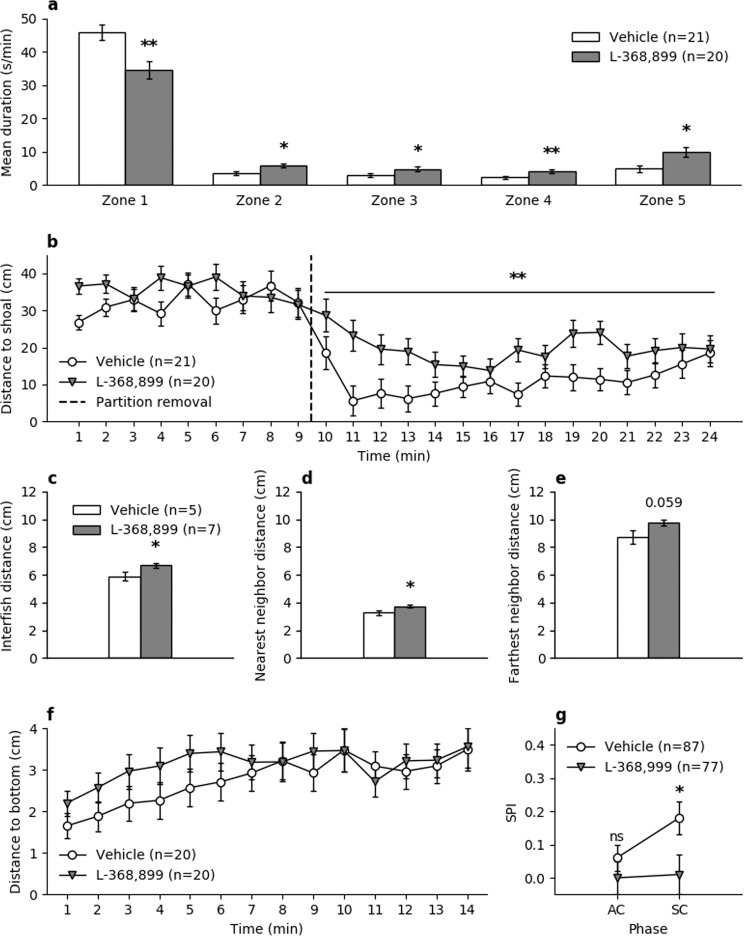
Social preference and anxiety-like behaviours in zebrafish. (**a**) Time spent in zones of the social preference test in zebrafish adults after acclimation, with Zone 1 being closest to the side tank where the stimulus shoal was placed. (**b**) Distance between adult focal fish and stimulus shoal in the social preference test, during the acclimation period (0–9 minutes) and test period (10–24 minutes) of the social preference test in adults. (**c–e**) Shoaling parameters during the shoaling test in adults. (**f**) Distance to bottom over time during the novel tank diving test in adults. (**g**) Social preference index (SPI) of the social preference test in larvae, during the acclimation (AC) phase and the social cue (SC) phase. All values are means ± standard error of the mean.

**Figure 4:**
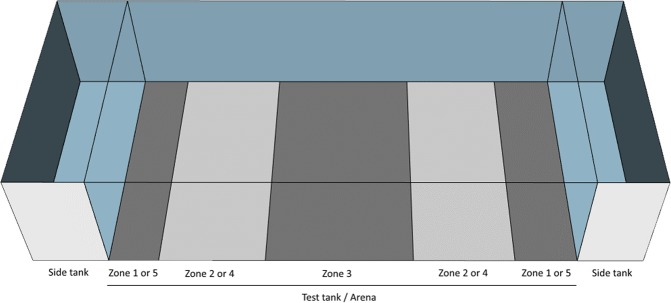
Experimental setup for social preference in adult zebrafish. The focal fish was placed in the test tank, and a stimulus shoal was placed in one of the side tanks, while the opposite side tank contained no fish. Visual barriers were placed between tanks and removed after the acclimation period was complete. Zones were designated as Zone 1 (the zone closest to the stimulus shoal) through to Zone 5 (the zone furthest away from the shoal).

The test used was similar to the social preference paradigm commonly employed in mice^47^. Individual focal fish was placed in a test tank, which in turn was situated between two side tanks, one contained a shoal of conspecifics and one was empty. L-368,899-treated zebrafish spent significantly less time in Zone 1 (closest to the shoal) in comparison to vehicle-treated fish (t(39) = 3.339, p = 0.0016), and significantly more time in all the other zones during the 15 minutes of test time (Fig. 3a; Fig. 4).

Antagonist-treated fish were significantly further away (distance from shoal) from the shoal in comparison to vehicle-treated fish during the 15 minutes of social test time (F(1,39) = 10.79, p = 0.0022; Fig. 3a; Fig. 4). However, both vehicle-treated and antagonist-treated fish responded to the visual stimuli of a conspecific shoal in a neighbouring tank by approaching the shoal (paired T-test comparing average distance to shoal during acclimation and test period; vehicle: t(20) = 10.544, p = 1.2e-9; antagonist: t(19) = 5.983, p = 9e-6).

### Shoaling in adult zebrafish

Shoaling data for shoals containing four fish was analysed for the following social parameters: average inter-fish distance (IFD)^25^, nearest neighbour distance (NND)^48^, and farthest neighbour distance (FND)^49^. Initial analyses revealed one vehicle-treated shoal as scoring more than two standard deviations above the mean for IFD and NND, and very close to two standard deviations above the mean for FND. Including this shoal in the analyses revealed no significant differences on any of the outcome parameters, but when excluded, the antagonist-treated shoals were significantly more spread out than vehicle-treated shoals (IFD: t(10) = 2.276, p = 0.046; NND: t(10) = 2.324, p = 0.042; FND: t(10) = 2.129, p = 0.059; see Fig. 3c–e).

### Novel tank diving in adult zebrafish

Anxiety-like behaviour in zebrafish is evident in a tendency to move towards the bottom when being in novel environments^50^. To study the role of zOT in anxiety, fish were placed in novel tanks and data was analysed for the following anxiety measures: average time (seconds) per minute spent in the top zone of the tank, latency (seconds) to enter the top zone, and average distance to bottom (cm) of the tank per minute. There were no differences between groups with regards to distance to bottom (F(1,38) = 0.522, p = 0.48; Fig. 3f), latency to first entry in top zone (t(38) = 0.605, p = 0.55), or average time spent in top zone (F(1,38) = 0.294, p = 0.59).

### Social preference in larval zebrafish

**Figure 5:**
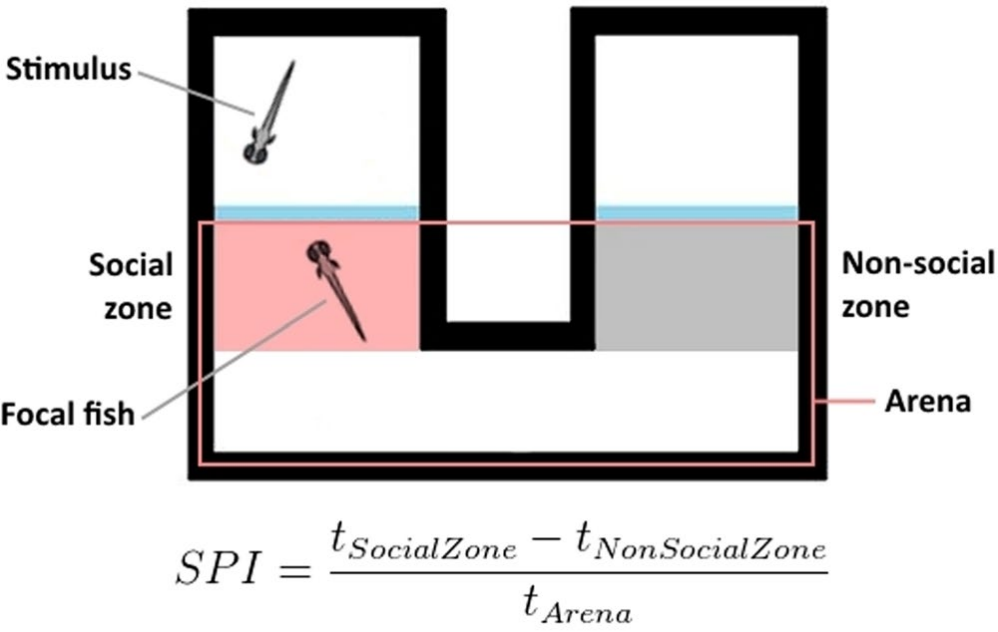
Experimental setup for social preference in 3-week-old zebrafish larvae. Social Preference Index (SPI) was calculated as the formula indicates, for the acclimation period and the social cue period separately.

The social preference test for larvae has been described elsewhere^27^, and is illustrated in Fig. 5. Briefly, focal larvae were placed in arenas where they could spend time close to a chamber with a stimulus larvae or close to an empty chamber (social cue period), following an acclimation period where no stimulus was present. A social preference index (SPI) was calculated for each of these periods, to determine preference for the chamber that would (acclimation period) or did (social cue period) contain the stimulus larvae. Vehicle-treated larvae displayed a significantly increased SPI from the acclimation period to the social cue period (t(86) = 2.093, p = 0.039). This increase in SPI from acclimation to social cue was not present in antagonist-treated larvae (t(76) = 0.177, p = 0.86), and the antagonist-treated larvae had significantly lower SPI during the social cue period, compared to vehicle-treated larvae (t(162) = 1,98, p = 0.049; see Fig. 3g).

## Discussion

Although recent studies suggest that exogenous OT ligands modulates social behaviour in adult zebrafish^42^, the relevance of endogenous zOT and its two receptor subtypes – Oxtr and Oxtrl – for social behaviour in adult and larval zebrafish remains to be clarified. Therefore, our initial pharmacological *in vitro* studies were primarily aimed to explore the effects of zOT and the OT receptor antagonist L-368,899 on the two zOT receptors as well as the two most closely related zVP receptors, encoded by the genes *avpr1aa* and *avpr1ab*. Intriguingly, the non-peptidergic OT receptor antagonist L-368,899, known to specifically inhibit the mammalian OT receptor^45,46^, potently antagonised both of the zOT receptors without reducing the effect of the peptide agonist on the zVP receptors. Thus, our behavioural studies using L-368,899 imply that endogenous zOT, and possibly zVP, regulates the types of social preference studied here through actions on either or both of the zOT receptors in both adult and larval zebrafish, but not (or less so) via the two VP-1 receptors. In addition, we found that the effect of zOT receptors on social preference was independent of anxiety-like behaviour.

In mammals, OT and VP act partly through each other’s receptors^38^. Therefore, we sought to understand how zOT and zVP act through the two zOT receptors (Oxtr and Oxtrl) and the two zVP receptors (Avpr1aa and Avpr1ab). We did not consider the four more distantly related V2 receptors^34,40^ because these belong to an earlier evolutionary branch and have not been demonstrated to be expressed in the brain, but rather have their primary roles in the regulation water and salt balance. While zOT acted on both of the zebrafish zOT receptors as well as the Avpr1ab with similar high potencies of EC50 around 3 nM (Table 1), zVP was found to be most potent on the Avpr1ab receptor (2.22 nM) with slightly lower potencies on the two zOT receptors. Hence, our results indicate that zOT and zVP can act through each other’s receptors. This is in line with what is known from other species, and is important to consider when interpreting the results from, for example, pharmacological studies using OT and VP. Unexpectedly, whereas neither of the investigated endogenous ligands seemed to be very potent on the Avpr1aa receptor, human OT exerted moderate agonistic activity on this receptor. This information may be useful for structural modelling of peptide binding to the different receptor subtypes. Due to the low potencies observed for both zOT (317 nM) and zVP (727 nM), the role of the Avpr1aa receptor may be to convey responses only under circumstances of very high peptide concentrations. The fact that this receptor has been identified in several teleost species, all of which are distantly related to the zebrafish^34^, indicates that it plays some important role that has maintained its existence throughout teleost evolution. This makes it interesting to investigate its role in zebrafish physiology.

Given the described specificity of L-368,899 for Oxtr and Oxtrl, this antagonist can be used as a tool for exploring to what extent the effects of zOT are mediated specifically by the zOT receptors, as opposed to the zVP receptors. Consequently, our behavioural studies – showing that L-368,899 inhibits social preference, and potentially also shoaling behaviour – provide evidence for an important role of zOT receptors in regulation of zebrafish social behaviour. However, as the antagonist did not completely abolish social preference, we cannot exclude the possibility that zVP or other molecules may also play a role in the neurobiology of zebrafish social behaviour^42,51^. Furthermore, as the antagonist blocked both of the zOT receptors, we cannot here draw any conclusions regarding the potentially different roles between them.

The zebrafish offers excellent opportunities for exploring group behaviour, as this species forms cohesive shoals, and grouping propensities can be readily quantified^52^. Therefore, we explored the effect of L-368,899 on shoal behaviour in zebrafish. Administration of the OT orthologue in birds, mesotocin, increases flock formation while a mesotocin antagonist reduces it^53^. The influence of the nonapeptides on group behaviour in zebrafish is thus far relatively understudied (however, see reports by Lindeyer *et al*.^42^ and Braida *et al*.^51^), but our results suggest a tentative and small effect of the OT receptor antagonist on shoaling in adult zebrafish.

Social preference develops during the first two to three weeks of a zebrafish’s life^26–28^. The underlying neurobiology of this development is unclear, although Dreosti and co-workers^27^ could inhibit social preference with an NMDA receptor antagonist and with high concentrations of ethanol. In the current study, we provided evidence that endogenous zOT plays a role in social behaviour of zebrafish larvae. Future studies should investigate to what extent zOT and its receptors contribute to the development of social behaviour in young zebrafish.

Distance between fish in a shoal is strongly dependent on context – for example, new environments and predatory threat^54^. In mammals, OT regulates anxiety and fear responses^5^, and pharmacological nonapeptide manipulation in adult zebrafish affects the response to predatory stimulus^42^. To understand if the effect of L-368,899 on social preference is mainly related to anxiety regulation or social attention, we investigated fish treated with antagonist or vehicle in the novel tank diving paradigm, an established test of anxiety-like behaviour^55^. As no differences in any of the anxiety parameters were detected, our results indicate that the two zOT receptors are involved in the regulation of social preference, independent of anxiety-like behaviour. Hence, our results suggested that the social effects of OT might be due to direct actions on the social attention neural network rather than caused by indirect actions on brain regions regulating anxiety.

In recent years researchers have started to elucidate the neurobiology of social preference. Thus far, dopamine^56^ and serotonin^57^ in the mesolimbic system and excitation-inhibition balance^58^ seem to play a prominent role in social investigation in rodents. Similarly, levels of dopamine and serotonin are modulated in socially interacting fish^59–61^ and social preference behaviour decreases after treatment with a dopamine D1 receptor antagonist^62^. Moreover, NMDA receptor antagonists inhibit social preference in both adult^44,49,63^ and larval^27^ zebrafish. In rodents, release of dopamine^64^, and serotonin^65^ as well as excitation-inhibition balance^66^ have been suggested as key mechanisms for the actions of OT on social behaviour. In zebrafish, zOT treatment rescued deficits in social preference induced by the NMDA receptor antagonist MK-801^44^, further indicating that glutamate neurotransmission, and tentatively the balance between excitation and inhibition, may be important for the actions of zOT on social behaviour. Future studies should evaluate to what extent these mechanisms are involved in the effects of zOT on social preference in zebrafish. In addition, rodents lacking the autism-causing genes CNTNAP2^67^ and SHANK3^68^, respectively, display aberrations in social behaviours, which are reversed by OT treatment. Interestingly, zebrafish mutants lacking CNTNAP2^69^ or SHANK3^70^ gene expression also display autism-like behaviours. Further studies are needed to understand if and how OT may regulate social interactions and other relevant behaviours in zebrafish mutants lacking CNTNAP2, SHANK3, and other genes contributing to the risk of autism.

In a study by Zimmermann and co-workers^44^, L-368,899 by itself did not decrease social preference in adult zebrafish. These opposing results between their study and ours are likely due to the rather stark difference in drug concentrations. Another study on zebrafish investigating the effects of zOT, zVP, and receptor antagonists, showed that zVP and the VP receptor antagonist decreased social preference whereas zOT and the OT receptor antagonist did not alter social preference^51^. Their results differ from the ones presented here; possibly due to differences in the experimental designs. For example, their strain of zebrafish was not specified, they used a peptidergic OT receptor antagonist with unknown receptor specificity, and they initiated the behavioural test five minutes after drug administration. Hence, it is difficult to compare their results with ours.

In conclusion, our results indicate that the two zOT receptors are involved in the regulation of social behaviour in adult and larval zebrafish, and that this effect is independent of anxiety-like behaviour. Moreover, our results together with the facts that zebrafish share many anatomical and physiological features with humans and have an archetypical vertebrate brain, as well as being cost-effective and amenable to genetic manipulation^71^, suggest that zebrafish offer an excellent opportunity to study the role of OT in social behaviour in vertebrates. In addition, our results show that L-368,899 is an appropriate tool for future studies of social and collective behaviours in zebrafish and presumably other species, although we recommend that the properties of the pharmacological tools be characterised using the cloned receptors before embarking on behavioural studies.

## Methods

### Pharmacological characterisation of zebrafish OT receptors and VP receptors

#### Preparation of the expression vector for receptors

The receptor constructs were ordered from GeneScript and subcloned into a pcDNA3.1/CT-GFP-TOPO vector. The coding sequences of the GenBank entries NM_001199370.1 (*oxtr*), NM_001199369.1 (*oxtrl*), NM_001301114.1 (*avpr1aa*), and NM_001297676.1 (*avpr1ab*) were used for synthesis. The stop codons were removed, and an additional ATP was added to facilitate hybrid protein expression with GFP in frame from the vector. The GCCACC Kozak sequence was inserted before the start codons. After transformation of the plasmid constructs into TOP10 competent cells (Thermo Fisher Scientific), the Maxiprep (Thermo Fisher Scientific) method was used to amplify the plasmids for transfection.

### Membrane expression of receptors

HEK293 cells were grown on coverslips treated with poly-D-lysine. The expression constructs of receptors, with GFP tagged on the C-terminal, were transfected using Lipofectamine 2000 (Thermo Fisher Scientific), then incubated for 24 hours at 37 °C, with 5% CO_2_. The medium was discarded and the coverslip with HEK293 cells was gently washed twice with PBS. Cells were then fixed with 95% ethanol for 10 minutes, and following this, coverslips were placed upside down onto slides containing a drop of Vectashield mounting medium (with DAPI), and sealed with nail polish. GFP expression pictures were produced using a confocal microscope.

### Inositol phosphate accumulation assay

The Inositol phosphate accumulation assay was performed according to previous descriptions to measure the potency of different ligands for zebrafish different oxytocin related receptors^72^. Each receptor construct was transfected into HEK293 cells using Lipofectamine 2000 (Thermo Fisher Scientific). Myo2-^3^H-inositol (PerkinElmer) at 3 μCi/mL was added the day after transfection. On day three, cells were re-suspended in assay buffer (10 mM LiCl, 20 mM HEPES, 137 mM NaCl, 5 mM KCl, 0.44 mM KH_2_PO_4_, 4.2 mM NaHCO_3_, 1.2 mM MgCl_2_, 1 mM CaCl_2_, and 10 mM glucose), after incubation in a PBS/EDTA mixture (0.2 g/L). After 10 minutes of pre-incubation at 37 °C, the cells were stimulated with serial dilution of peptides for 25 minutes at 37 °C. To determine the IC50 value of the antagonist, L-368,899, a serial dilution (−11 to −4 log M) was added together with 1 μM zVP (vasotocin) to the cells. 1 μM zVP could generate the maximum response for all receptor subtypes. Cells were lysed for 60 minutes at 4 °C with an equal volume of 0.8 M perchloric acid, and then neutralised with KOH/KHCO_3_ solution. Ion exchange chromatography on AG 1-X8 resin (Bio-Rad) was used to collect the generated ^3^H-inositol phosphates. The resin was washed with buffer containing 5 mM Na_2_B_4_O_7_ and 60 mM NH_4_-formate and eluted with buffer containing 1 M NH_4_-formate and 0.1 M formic acid. Samples were mixed with OptiPhase HiSafe (PerkinElmer Waltham, MA, USA) and ^3^H radioactivity was measured with a Tri-Carb 2910TR liquid scintillation counter (PerkinElmer Waltham, MA, USA). The assays were performed in duplicate for each concentration and repeated at least 3 times.

### Zebrafish behaviour

#### Husbandry

Zebrafish (*Danio rerio*) of AB line were bred and housed at the University of Gothenburg. Adults were housed in an automated standalone system (Aquaneering, San Diego, USA), supplied by deionised water, and supplemented with Tropic Marin Pro Reef salt (Tropic Marin, Wartenberg, Germany) and NaHCO_3_ to achieve a conductivity of 700 μS and a pH range of 7.2–7.4. Larvae were raised in freestanding tanks, and the same water parameters were applied. Adults and larvae were held under a 14:10 hour light:dark cycle (8 AM–10 PM), at a temperature of 27–28 °C. Adults were fed twice a day with a mixture of Tetra Pro crisps flake food (Tetra Fish, Melle, Germany) and granular pellets (ZM Fish foods, Winchester, UK). Larvae were fed three times per day with granulated fry food (ZM-000, ZM-100, ZM-200, ZM Systems, Winchester, UK) from 6 days post fertilisation (dpf), and brine shrimp nauplii (*Artemia salina*, ZM Fish foods, Winchester, UK) from 10 dpf. Adult fish were also fed hatched brine shrimp nauplii once a day. All larval experimental work took place at three weeks of age, and all animals used in both adult and larval experiments were experimentally naïve. Experiments adhered to a study protocol approved by the animal ethics committee of Gothenburg, and followed the guidelines of the Swedish National Board for Laboratory Animals.

### Experimental protocols for adult and larval studies

Adult and larval behavioural testing was performed between 9 AM and 4 PM. All fish were fed prior to and after experiments, and larvae were also given an additional feed at midday. Adult fish were of a 1:1 sex ratio, and focal fish and stimulus fish were size-matched. Adult fish and shoals were filmed using a web camera and Debut Video Capture Software (NCH Software, Greenwood Village, USA), and videos were analysed using EthoVision version 11 (Noldus, Wageningen, the Netherlands).

### Social preference in adult zebrafish

The experimental setup comprised one large test tank (60 cm × 30 cm), and two smaller side tanks (15 cm × 30 cm) positioned at the short ends of the test tank; see Fig. 4. The test tank (the arena) was divided into five different zones used when tracking the video recordings, see Fig. 4. The zone closest to the stimuli shoal was always designated Zone 1, and the zone furthest away from the shoal was always designated Zone 5. The water in the tanks matched parameters for housing conditions and had a depth of 10 cm. All tanks were visually isolated from each other during the acclimation period of 10 minutes.

A stimulus shoal of eight zebrafish, with a 1:1 sex ratio, was placed in one of the side tanks, while the opposite side tank remained empty. Focal fish and shoal fish were selected to be of similar size in order not to evoke adverse reactions, such as anxiety or aggression. The experiments were thoroughly randomized between treatment groups for time of day and shoal used. The stimulus fish had been habituated to the room and aquarium for 24 hours prior to the start of the experiments. Two shoals were used as stimulus shoals throughout the experiment. The side for the stimulus shoal was alternated in order to avoid any potential place preference of the focal fish. The reasons for using two shoals for all focal fish were to decrease the behavioural variation in focal fish that may be caused by inter-shoal variations, to decrease the stress in stimulus fish caused by netting and transferring them between tanks that may be transferred to the focal fish, and to limit the number of fish used according to the 3 R principles. There were no statistical significant effects of day of testing (One-way ANOVA; p > 0.7), time of testing (One-way ANOVA; p > 0.5), or the identity of the stimulus shoal (shoal 1 versus shoal 2; un-paired t-test; p > 0.1) on the total time the focal fish spent in Zone 1 (closest to the shoal) during the 15 minutes test time.

At 24 hours prior to the experiment, zebrafish were weighed, placed into separate holding tanks, and brought to the experimental room in order to acclimatise. The following day fish were intraperitoneally injected with vehicle Ringer’s solution (13 females and 8 males), or the antagonist (11 females and 10 males). The Ringer’s solution recipe was retrieved from the Zebrafish Information Network (ZFIN, University of Oregon, Eugene, OR, USA). The non-peptidergic OT receptor antagonist (L-368,899 hydrochloride; Tocris Bioscience, Abingdon, United Kingdom) had a working concentration of 10 µg/µl, and the injected volume was corrected to the mass of the fish (10 µl per gram of the fish). Pilot experiments using 5 and 50 μg L-368,899 per gram fish did not affect social preference; therefore 100 µg per gram fish was used.

Intraperitoneal injections were conducted using a 1 mL U-100 BD MicroFine™ Insulin Syringe (Becton Dickinson France S.A.S., France). Post injection, animals were placed into their holding tanks for one hour to recover. The decision to wait one hour between injection and behavioural testing was based on previous studies showing that L-368,899 has a half-life of about 2 hours^73^. Furthermore, studies in monkeys^45^ and rodents^74^ prove robust behavioural effects one hour after treatment with L-368,899. Following this, focal fish were individually placed in the middle of the test tank and left to freely move around for ten minutes (acclimation period) before visual partitions were removed, which initiated the start of the test period (15 minutes). The first minute of the acclimation period has been removed in order to achieve optimal tracking in EthoVision, hence the reported results for the acclimation period is over nine minutes.

### Shoaling test in adult zebrafish

The shoaling test was performed to examine the effects of L-368,899 on social group behaviour, a test widely used to assess shoaling sensitivity to various drugs and experimental settings^75,76^.

The experimental setup comprised a 1.8 L trapezoidal tank (height 16 cm × length at the top 26 cm × length at the bottom 23 cm × width 6 cm, Aquaneering, San Diego, USA). Fifty-six zebrafish were randomly grouped into shoals of four (two males and two females) three days prior to the experiment. One member of a vehicle-treated shoal died during this pre-experimental period, which resulted in the exclusion of that shoal. On day four all shoal members were weighed and injected with either fish Ringer’s (n = 6 shoals) or L-368,899 (n = 7 shoals), using the same procedure described above. Shoals were returned to their respective holding tanks for one hour to recover, and then gently transferred to test tanks, in which they were filmed for 15 minutes.

### Novel tank diving in adult zebrafish

A novel tank diving test was executed individually in order to examine the effect of the antagonist on anxiety in zebrafish. Anxiety-like behaviour in zebrafish is evident in a tendency to move towards the bottom of novel environments^50^. The novel tank diving test used here has been described elsewhere^55^. In brief, the same procedure as for the shoaling test described above was used, except that fish were housed in groups of 4–6 individuals 24 hours prior to the experiment in order to keep netting and potential stress to a minimum, and each fish was placed the test tank individually. A total of 20 vehicle-treated fish and 20 antagonist-treated fish were tested.

### Social preference in larval zebrafish

The social preference test for young zebrafish has been described elsewhere^27^. In brief, experiments were performed in custom-built arenas; see Fig. 5, consisting of laser-cut 5 mm high white acrylic walls placed on transparent flooring with transparent acrylic partitions. The dimensions of each arena were 40 × 32 mm, and the chambers containing stimulus fish was 15 × 15 mm. The areas in front of these stimulus compartments were designated Social Area (where the stimulus was placed) or Non-Social Area (where the stimulus was not placed). The water depth was 5 mm, and water parameters matched the ones housing water parameters. Nine arenas were placed adjacent to each other on an infrared (IR) back-light diffusing both IR and white light (Noldus, Wageningen, the Netherlands), with a digital monochrome high resolution GigE camera overhead (Noldus, Wageningen, the Netherlands), and surrounded by an opaque enclosure.

A 10 mM stock solution was prepared by dissolving L-368,899 in H_2_O (Milli-Q water purification system), and subsequently stored at −20 °C. The stock solution was diluted to a working concentration of 100 µM by addition of water adjusted to the same parameters kept in the fish facility. Larvae were immersed in 100 µM L-368,899 solution (treated fish) or in water matching housing conditions (control fish) for 1 hour prior to testing. Focal larvae were then transferred to test arenas and filmed for 15 minutes, with no stimulus present (acclimation period; AC). Following this, one stimulus larva was placed in a randomly selected stimulus chamber of each arena, and focal larvae were filmed for another 15 minutes (social cue period; SC). A total of 137 vehicle-treated fish and 109 antagonist-treated fish were tested, and after using the exclusion criteria described below, 87 vehicle-treated fish and 77 antagonist-treated fish were included in the statistical analyses.

### Data analysis

All data for the social preference and novel tank diving experiments in adults were extracted as processed means per minute from EthoVision. For the shoaling experiments, raw data was exported from EthoVision, and then post-processed by an in-house script written in Python 3.4, in order to compute IFD, NND, and FND as described elsewhere^49^. For timelines, repeated measures analysis of variance (RM-ANOVA) was used. For pairwise comparisons of total means between groups, Student’s T-tests were used.

Larval results were analysed using a social preference index (SPI). SPI was calculated by subtracting the time a focal larva spent in the Non-Social Area from the time it spent in the Social Area, and then dividing the result by the total time the larva was tracked in the entire arena. This resulted in an index ranging from +1 (a larva spent all its time in the Social Area) to −1 (a larva spent all its time in the Non-Social Area), with 0 indicating no preference. SPI was calculated for each focal larva for the AC period and the SC period separately. Individuals were excluded if they did not explore both arms of the arena during the AC period, or if they were not tracked for >10% of either or both of the periods. All data was analysed using paired T-tests to investigate change within the control and treated groups, and Student’s T-tests to investigate difference between the groups for each phase.

Results are presented as mean values ± standard error of mean (SEM). All analyses were performed using the statistical software SPSS 22, with an alpha of <0.05.

## Supplementary information

Supplementary Data S1.

## Data Availability

The script used to extract shoaling parameters is available in the Supplementary Information as Supplementary Data S1.
