# Supplementary material for: Oxytocin Receptors Regulate Social Preference in Zebrafish
Source: Sci Rep. 2020 Mar 25;10:5435. doi: 10.1038/s41598-020-61073-4 (PMC7096398; doi:10.1038/s41598-020-61073-4)
Supplement: Supplementary file 1 — Supplementary Data S1. [file 41598_2020_61073_MOESM1_ESM.pdf]

Oxytocin Receptors Regulate Social Preference in Zebrafish

Jenny Landin[1], Daniel Hovey[1], Bo Xu[3], David Lagman[3], Anna Zettergren[2], Dan Larhammar[3], Petronella Kettunen[2]#, Lars Westberg[1]#*

[1]Department of Pharmacology, Institute of Neuroscience and Physiology, University of Gothenburg, Sweden

[2]Department of Psychiatry and Neurochemistry, Institute of Neuroscience and Physiology, University of Gothenburg, Sweden

[3]Department of Neuroscience, Unit of Pharmacology, Science for Life Laboratory, Uppsala University, Sweden

#Equal author contribution

*Corresponding author: Lars Westberg

E-mail: lars.westberg@pharm.gu.se

Phone: +46-(0)31-786 3431

Postal address: Department of Pharmacology, Institute of Neuroscience and Physiology, Sahlgrenska Academy, University of Gothenburg, Box 431, SE-405 30 Gothenburg, Sweden

Supplementary Data S1: The script used to extract shoaling parameters

```
import pandas as pd

import numpy as np

import xlrd

import glob


# Create Fish object for each fish in a shoal of 4 fish, containing all

# necessary information neatly packaged after initial cleanup.

class Fish(object):

			def __init__(self, df):

						self.df = pd.DataFrame(data=df, copy=True)

						self.trialid = self.df.loc[df["Number of header lines:"] == "Trial name"]["35"].item().split()[1]

						self.arenaid = self.df.loc[self.df["Number of header lines:"] == "Arena name"]["35"].item().split()[0]

						# Create a shoal id in order to specify the statistial unit.

						self.shoalid = self.trialid # + self.arenaid

						self.group = self.df.loc[self.df["Number of header lines:"] == "Treatment"]["35"].item()

						self.subjectid = self.df.loc[self.df["Number of header lines:"] == "Subject name"]["35"].item()

						# Locate the actual header line for variables of interest

						self.headeridx = self.df[self.df["Number of header lines:"] == "Trial time"].index.item()

						self.parse(self.df)

						self.nnd = pd.DataFrame(data=self.df["NND"], copy=True)

						self.fnd = pd.DataFrame(data=self.df["FND"], copy=True)

						self.ifd = pd.DataFrame(data=self.df["IFD"], copy=True)

						self.dist = pd.DataFrame(data=self.df.iloc[:,0:3], copy=True)

						# Create variables containing number of "connections" from this fish to others, i.e. number of distances

						# that fall below the cutoff bl (body length) - later used in the compile function.
```

```
			self.connect = self.dist[self.dist < bl].count(axis=1)
		# Define function for general parsing and cleanup, to get variables that will later be
averaged
		# across the shoal.
		def parse(self, messy):
			messy.columns = messy.iloc[self.headeridx]
			messy.drop(messy.index[0:(self.headeridx+2)], inplace=True)
			exclude, keep = [], []
			for i in messy.columns.tolist():
				if "Recording time" in i:
					keep.append("Time")
				elif "Distance between subjects" in i and self.subjectid in
i:
					exclude.append(i)
				elif "Distance between subjects" in i:
					if int(self.subjectid.split()[1]) <
int(i.split()[5]):

		keep.append(self.subjectid.split()[1] + "-" + i.split()[5])
					else:
						keep.append(i.split()[5] + "-"
+ self.subjectid.split()[1])
				else:
					exclude.append(i)
			for i in exclude:
				messy.drop(i, axis=1, inplace=True)
			messy.columns = keep
			messy.reset_index(drop=True, inplace=True)
			messy.replace("-", np.NaN, inplace=True)
			messy["NND"] = messy[messy.columns[1:]].min(axis=1)
			messy["FND"] = messy[messy.columns[1:4]].max(axis=1)
			messy["IFD"] = messy[messy.columns[1:4]].mean(axis=1)
			messy.set_index(["Time"], drop=True, inplace=True)
```

```
# Create function to compile shoal level data.
def compile(shoal):
    sid = shoal[0].shoalid
    gr = shoal[0].group
    outcomes = []
    # Average and resample (per minute) each outcome variable.
    for outcome in ["nnd", "fnd", "ifd"]:
        # Create list of dataframe objects that can be combined and then later operated on.
        dfconv = []
        for f in shoal:
            if outcome == "nnd":
                dfconv.append(f.nnd)
            elif outcome == "fnd":
                dfconv.append(f.fnd)
            elif outcome == "ifd":
                dfconv.append(f.ifd)
        combo = pd.concat(dfconv, axis=1)
        combo["Avg"] = combo[combo.columns].mean(axis=1)
        combo.drop(combo.columns[:4], axis=1, inplace=True)
        combo.set_index(pd.to_datetime(combo.index * 1000, unit="ms"), inplace=True)
        avgmin = combo.resample("T").mean()
        minutes = []
        for m in range(len(avgmin)):
            minutes.append("Min" + str(m+1))
        avgmin["Minute"] = minutes
        avgmin["Outcome"] = outcome
        outcomes.append(avgmin)
    # Calculate shoal index and resample per minute.
    dfconv = []
```

```
for f in shoal:
        dfconv.append(f.connect)
combo = pd.concat(dfconv, axis=1)
# Below rather clunky method for defining shoal index depending on how many connections exist in total,
# hopefully covering all possible combinations of fish connections. NB only works with 4 fish in a shoal.
# Shoal index should be considered unreliable.
conditions = [
        (combo.eq(3).any(1)),
        (combo.sum(axis=1)>=7),
        (combo.sum(axis=1)==6) & (~combo.eq(0).any(1)),
        (combo.sum(axis=1)==6) & (combo.eq(0).any(1)),
        (combo.sum(axis=1)==4),
        (combo.sum(axis=1)==2),
        (combo.sum(axis=1)==0),
        ]
choices = [4, 4, 4, 3, 3, 2, 1]
combo['Avg'] = np.select(conditions, choices)
combo.drop(combo.columns[:4], axis=1, inplace=True)
combo.set_index(pd.to_datetime(combo.index * 1000, unit="ms"), inplace=True)
avgmin = combo.resample("T").mean()
minutes = []
for m in range(len(avgmin)):
        minutes.append("Min" + str(m+1))
avgmin["Minute"] = minutes
avgmin["Outcome"] = "si"
outcomes.append(avgmin)
# Combine all outcome variable dataframes into one - long format.
combined = pd.concat(outcomes, axis=0)
combined["ShoalID"] = sid
combined["Variable"] = combined["Outcome"] + "." + combined["Minute"]
```

```
        combined.drop("Minute", axis=1, inplace=True)
        combined.drop("Outcome", axis=1, inplace=True)
        # Finally pivot everything to wide format.
        pivoted = combined.pivot(index="ShoalID", columns="Variable", values="Avg")
        # Add mutant yes/no variable.
        pivoted["Treatment"] = gr
        return pivoted

# Define function to handle raw data file level data, parsing a file with an unspecified number of shoals.
def parse(imp):
        sheets = imp.sheet_names
        fish = []
        shoallist = []
        shoals = []
        out = []
        for sheet in sheets:
                fish.append(Fish(imp.parse(sheet)))
        for f in fish:
                if f.shoalid not in shoallist:
                        shoallist.append(f.shoalid)
                        shoals.append([])
        for i,s in enumerate(shoallist):
                for f in fish:
                        if f.shoalid == s:
                                shoals[i].append(f)
        for shoal in shoals:
                out.append(compile(shoal))
        return pd.concat(out, axis=0)

# Main function to handle all files in the location of the script,
```

```
# with reasonably helpful printed progress rate and current file.


def main():
		summary = []
		files = glob.glob("*.xlsx")
		total = len(files)
		count = 0
		for file in files:
				print("Parsing " + file)
				# Below was added because glob creates a list with a file that doesn't
exist, halting everything.
				try:
						summary.append(parse(pd.ExcelFile(file)))
						count += 1
						print(str(int((count/total)*100)) + "% done.")
				except:
						print("Failed.")
						continue
		final = pd.concat(summary, axis=0)
		final["nndAvg"] = final["nndAvg"] = final[[col for col in final.columns if "nnd." in col and
int(col.split(".")[1].strip("Min")) <= 15]].mean(axis=1)
		final["fndAvg"] = final["fndAvg"] = final[[col for col in final.columns if "fnd." in col and
int(col.split(".")[1].strip("Min")) <= 15]].mean(axis=1)
		final["ifdAvg"] = final["ifdAvg"] = final[[col for col in final.columns if "ifd." in col and
int(col.split(".")[1].strip("Min")) <= 15]].mean(axis=1)
		final["siAvg"] = final["siAvg"] = final[[col for col in final.columns if "si." in col and
int(col.split(".")[1].strip("Min")) <= 15]].mean(axis=1)
		return final


# Specify body length cutoff for the shoal index calculations.
bl = 3.2
# Run main function and output immediately to result file, ready to import to SPSS.
main().to_excel("result.xlsx")
```
